# EEG Neurofeedback for Major Depressive Disorder: A Scoping Review

**DOI:** 10.31083/AP50633

**Published:** 2026-06-25

**Authors:** Dan Cătălin Oprea, Jonathan Rabinowitz, Bogdan Gireadă, Bogdan-Ionel Tamba, Romeo-Petru Dobrin, Eliza-Mihaela Cămănaru, Vlad Teodor Iacob, Ana Caterina Cristofor, Alexandra Boloș, Roxana Chiriță

**Affiliations:** ^1^Grigore T. Popa University of Medicine and Pharmacy of Iasi, 700115 Iasi, Romania; ^2^Institute of Psychiatry “Socola”, 700282 Iasi, Romania; ^3^Bar Ilan University, 5290002 Ramat Gan, Israel; ^4^Department of Pharmacology, Clinical Pharmacology and Algesiology, Grigore T. Popa University of Medicine and Pharmacy of Iasi, 700454 Iasi, Romania

**Keywords:** neurofeedback, electroencephalography, biofeedback, major depressive disorder, endogenous depressions, depressive disorder

## Abstract

Neurofeedback (NF) is an emerging treatment for Major Depressive Disorder (MDD). NF is a training approach that enables individuals to self-regulate brain activity patterns associated with emotional and cognitive dysfunction. While interest is increasing, research results vary, and clinical implementation does not follow standardized protocols. This scoping review aimed to map and synthesize the existing peer-reviewed evidence on electroencephalography (EEG)-based neurofeedback interventions for MDD, with a focus on training protocols, neurophysiological targets, reported clinical outcomes, and methodological limitations. A systematic scoping review was conducted in accordance with Preferred Reporting Items for Systematic Reviews and Meta-Analyses Extension for Scoping Reviews (PRISMA-ScR) guidelines (OSF registration): https://doi.org/10.17605/OSF.IO/NQ3BW. Electronic searches were performed across Embase, PubMed, Web of Science, and ClinicalTrials.gov. Studies were included if they evaluated EEG-based neurofeedback interventions in adults with MDD and reported clinical or neurophysiological outcomes. Of 1299 records identified, 11 studies met the inclusion criteria, encompassing outcome data from 175 patients who received active EEG-NF. The studies observed a reduction in depressive symptoms following neurofeedback, most commonly using frontal alpha asymmetry protocols (k = 5). Potential improvements in cognitive performance were reported in 4 studies, particularly those incorporating upper-alpha or beta/theta modulation. Connectivity-based approaches using low-resolution electromagnetic tomography Z-score neurofeedback (low-resolution electromagnetic tomography (LORETA); k = 2) suggested possible effects on deep brain networks involved in emotional regulation, though replication remains limited. This scoping review outlines the existing evidence on EEG-based neurofeedback in major depressive disorder, highlighting the diversity of protocols, targets, and reported outcomes. While preliminary findings suggest potential clinical relevance, substantial methodological limitations and heterogeneity remain. These observations emphasize the need for more rigorous, standardized, and longitudinal research to better define the role of EEG-based neurofeedback in the treatment of MDD.

## Main Points

1. Electroencephalography (EEG)-based neurofeedback has been explored as a potential intervention for Major Depressive Disorder (MDD) across diverse protocols and neurophysiological targets.

2. Frontal alpha asymmetry was the most commonly used approach, while beta/theta and connectivity-based methods were less frequently applied.

3. Studies generally reported short-term, preliminary improvements in depressive symptoms, although methodological heterogeneity and a lack of controlled designs limit conclusions regarding actual therapeutic efficacy.

4. Small sample sizes, lack of sham-controlled designs, and limited follow-up represent key gaps in the current literature.

5. Further standardized and longitudinal research is needed to clarify the clinical role of EEG neurofeedback in MDD.

## 1. Introduction

Major depressive disorder (MDD) is a common mental disorder. Globally, depression affects approximately 5% of adults, with women experiencing it more frequently than men. In 2019 the economic cost of MDD globally was estimated at 1 trillion US dollars [1]. The World Health Organization projection estimates that depression will be leading cause of disease burden globally by 2030 [2].

Multiple hypotheses try to explain MDD pathogenesis including the hypothalamic‒pituitary‒adrenal axis dysfunction hypothesis, the monoamine hypothesis, the inflammatory hypothesis, the genetic and epigenetic anomaly hypothesis, the structural and functional brain remodeling hypothesis, and the social psychological hypothesis, but due to the complexity of the phenomena, none of the above can fully explain the pathogenesis [3,4,5,6,7,8,9,10].

The armamentarium for treating MDD includes psychotherapy, antidepressant medication [11], electroconvulsive therapy, and transcranial magnetic stimulation [12,13,14,15,16]. Additional treatment options are needed as approximately 30% of people treated for major depression do not respond to treatment.

Neurofeedback (NF) is an emerging therapeutic approach that establishes a brain–computer interface, providing patients with real-time feedback during treatment. This technique relies on Pavlovian operant conditioning, allowing individuals to perceive specific neuronal events through visual or auditory representations of their brain activity while engaging in cognitive tasks. The primary objective is to modulate brain waves or rhythms within targeted areas, thereby optimizing cognitive strategies for neuromodulation [17,18]. Essentially, NF brings unconscious processes into conscious awareness using various stimuli, enabling individuals to exert control over them.

NF uses different methods for monitoring neural activity, including electroencephalography (EEG) [19], functional magnetic resonance imaging (fMRI) [20], functional near-infrared spectroscopy (fNIRS) [21], and hemoencephalography (HEG) [22].

EEG-NF involves placing electrodes on the scalp to capture electrical brain activity, which is then recorded, processed, and presented visually or auditorily, often through metaphoric representations such as a car race or a melody. When the patient maintains brain waves within predefined parameters, positive reinforcement is provided (e.g., the car accelerates or the music plays correctly). Over successive sessions, difficulty levels are gradually increased [17,23,24]. Each session typically lasts between 20 and 25 minutes following equipment setup [25].

Compared to EEG-based neurofeedback, fMRI, fNIRS, and HEG rely on indirect measures of neural activity (BOLD or hemoglobin oxygenation), offering varying trade-offs between spatial and temporal resolution, but are generally limited by higher cost, lower temporal precision, and reduced practicality in clinical settings [26,27,28].

Given considerations such as cost, portability, practicality, and invasiveness, EEG-NF remains the most widely preferred method compared to other neuroimaging techniques [29], having the potential to be used as an add-on therapy.

Current research on EEG-based neurofeedback for major depressive disorder reveals several important scientific gaps that limit its clinical translation. The existing literature is characterized by a scarcity of rigorously designed sham-controlled randomized trials, making it difficult to distinguish genuine neurophysiological effects from placebo or expectancy-driven improvements [30,31]. Furthermore, considerable heterogeneity in NF protocols, including EEG targets, frequency bands, electrode placements, session duration, and training dose, further hampers reproducibility and cross-study comparability [32]. The underlying mechanisms remain poorly understood, as clinical improvements do not consistently align with the modulation of intended EEG targets [18], and baseline predictors of treatment response are rarely examined, limiting progress toward personalized NF interventions [33].

Together, these gaps underscore the need for larger, methodologically robust studies with standardized protocols, mechanistic outcome measures, and longitudinal follow-up to clarify the therapeutic potential of EEG-based NF in MDD.

This context highlights the need for a systematic mapping of the existing evidence to clarify which electroencephalography-based neurofeedback protocols and training approaches have the capability of translating neurophysiological modulation into clinically meaningful benefits for individuals with major depressive disorder.

EEG-NF was selected as the exclusive focus of this scoping review because it is the most widely implemented neurofeedback modality in clinical and community settings, relatively affordable, technically accessible, and generally well accepted by patients. Moreover, EEG-NF is increasingly promoted as a “revolutionary” intervention for depression in both clinical and commercial contexts, often without adequate differentiation between evidence-based protocols and speculative applications. In light of this rapid dissemination and commercialization, a critical and structured appraisal of the scientific evidence is warranted. Accordingly, this scoping review aims to systematically map the existing EEG-NF protocols and outcomes in MDD to identify clinically relevant approaches and highlight areas requiring further standardization and validation in the use of EEG-based neurofeedback as an intervention for major depressive disorder, with a particular focus on the characteristics of the applied protocols, targeted EEG biomarkers, and the range of reported clinical and cognitive outcomes.

## 2. Methods

### 2.1 Study Design

This study was conducted as a scoping review to comprehensively map the current evidence base regarding the efficacy of EEG-based neurofeedback interventions for individuals with major depressive disorder. This design was selected in light of the marked heterogeneity across the included studies, particularly with respect to neurofeedback protocols (targeted frequency bands, electrode placements and training duration), study designs, and the wide range of clinical and neurophysiological outcomes assessed.

The five-stage scoping review methodological framework proposed by Arksey and O’Malley was adopted to support comprehensive mapping of the available literature [34]. This approach was selected due to the heterogeneity of study designs and the limited number of high-quality trials in this research area. The scoping review was conducted in accordance with the PRISMA-ScR guidelines (**Supplementary Material**), ensuring a systematic, transparent, and reproducible process [35]. The scoping review protocol was preregistered with the Open Science Framework (https://doi.org/10.17605/OSF.IO/NQ3BW).

### 2.2 Research Question

The guiding research question was: What types of EEG-based neurofeedback interventions have been investigated in adults with major depressive disorder, and what clinical outcomes have been reported across studies?

The research question was informed by the Population–Concept–Context (PCC) framework [35,36]. The population comprised adults diagnosed with major depressive disorder. The concept encompassed EEG-based neurofeedback interventions, irrespective of protocol type or training parameters. The context was intentionally broad, including any clinical, research, or community setting, with no restrictions on geographic location, healthcare system, or cultural context. Because of the paucity of primary sources and the emerging nature of EEG-NF research in MDD, we also included case series and case reports to capture the full scope of available evidence. All included studies must have used standardized diagnostic tools (DSM or ICD criteria) to confirm the diagnosis. Reviews, editorial reports and conference abstracts were excluded.

### 2.3 Search Strategy

The literature search was systematically conducted across three major electronic databases: PubMed, Embase, Web of Science, and one trial registry: ClinicalTrials.gov (https://clinicaltrials.gov/). The search strategy was designed to capture the core elements of the research question, focusing on EEG-based neurofeedback interventions in adults with major depressive disorder. Searches included all relevant records published up to December 2025. No language restrictions were applied.

A comprehensive search strategy was developed using a combination of controlled vocabulary terms (MeSH) and free-text keywords, connected using Boolean operators (AND, OR). The primary concepts included EEG neurofeedback and depression. Database-specific adaptations of the search terms were applied to accommodate indexing differences across platforms.

The full PubMed search strategy was as follows:

((EEG Neurofeedback) OR (Brainwave Biofeedback) OR (EEG Feedback) OR (Electroencephalography Biofeedback) OR (Brainwave Feedback)) AND ((Major Depressive Disorders) OR (Clinical Depression) OR (Unipolar Depression)).

Equivalent search strategies were adapted for Embase, and Web of Science using their respective controlled vocabularies and indexing systems. In addition, ClinicalTrials.gov was searched using the parameters Condition/Disease: Depression and Intervention: EEG Neurofeedback to identify completed, ongoing, or unpublished trials.

### 2.4 Study Selection and Data Extraction

**Fig. 1.**
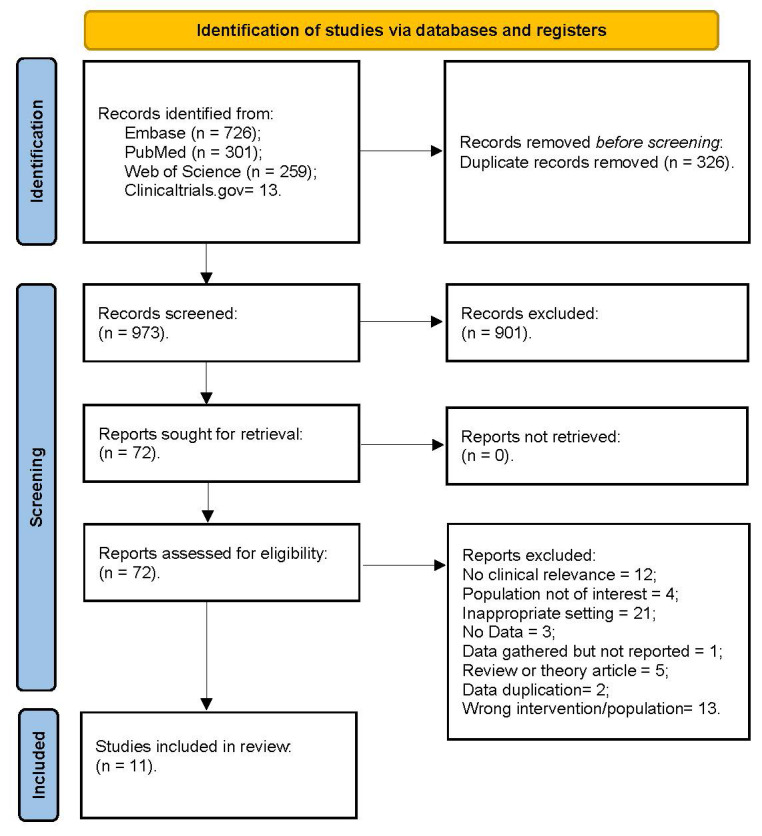
**PRISMA flowchart**. PRISMA, Preferred Reporting Items for Systematic Reviews and Meta-Analyses.

All records retrieved from the database searches were exported and consolidated using the HubMeta systematic review platform, which was employed to support reference management, duplicate removal, and screening procedures [37]. The initial search yielded a total of 1299 records across the databases. Following automated and manual deduplication, 326 duplicate records were identified and removed, leaving 973 unique articles for title and abstract screening. The initial screening was conducted independently by two authors (ODC and BG) based on the predefined inclusion and exclusion criteria. No language restrictions were applied at this stage. Discrepancies were resolved through discussion, and when necessary, by consultation with the full research team. Based on title and abstract screening, 901 records were excluded due to irrelevance to EEG-based neurofeedback, non-depressive populations, non-clinical designs, or theoretical focus. This process resulted in 72 articles eligible for full-text review. Full-text assessment was subsequently performed to determine final eligibility. Of these, 61 studies were excluded for reasons detailed in Fig. 1. Following this two-stage screening process, 11 studies and reports met the inclusion criteria and were retained for qualitative synthesis.

Data extraction was performed using a standardized chart that included study design (protocol, randomization, blinding, number and duration of sessions, follow-up), sample characteristics (size, sex, age, medication status), measures used, and key findings (Table 1, Ref. [38,39,40,41,42,43,44,45,46,47,48]).

**Table 1.** **Overview of included studies**.

References	Design 1. Protocol 2. Randomized (Y/N) 3. Blinded 4. Number of sessions 5. Duration of a session 6. Follow up	Sample 1. Number (sex) 2. Age 3. Medication (Y/N)	Measures used	Most relevant findings
Randomized controlled studies				
Choi et al. (2011) [38]	1. ALAY 2. Y 3. N 4. 10 5. 24 minutes 6. 1 month after	1. 12 NF vs 11 placebo 2. 28.46 ± 9.69 vs 28.54 ± 6.84 3. N	Daily Stress Scale; ATQ-P; ATQ-N; BDI-II; HAM-D; SFT; PFT; ST; EEG; EOG.	Increased right frontal alpha power and asymmetry scores (eyes-open only). Decreased depressive symptoms and enhanced executive function. 50% showed clinically meaningful improvement vs. placebo.
Wang et al. (2016) [40]	1. ALAY 2. Y 3. N 4. 6 5. 1 h 6. N	1. 7 NF vs 7TAU 2. 49.86 ± 3.98 vs 47.43 ± 13.84 3. Y	BDI-II; BAI.	A1 asymmetry index decreased in the control group but increased in the NFB group post-intervention. Responders showed reduced anxiety and depression scores after intervention. Non-responders exhibited increased anxiety and depression scores post-intervention.
Non-randomized control studies				
Escolano et al. (2014) [42]	1. UA increase at P3, Pz, P4, O1, and O2 2. N 3. N 4. 8 5. 20 min 6. N	1. 40 NF vs 20 NC 2. 53.70 ± 10.87 vs 49.50 ± 10.18 3. Y	EEG; HAM-D; BDI-II; PASAT; PHQ-9 RAVLT; STROOP; TMT; FAS.	Medium-large size effect on WM and processing speed with improvement for the NF group in comparison with the control group. Increase in power of UA across parieto-occipital lobes in task related activities.
Lee et al. (2019) [44]	1. 1 beta at T3 and A/T training; 5 beta at F3; 6 SMR at T4 and A/T training 2. N 3. N 4. 12–24 5. 1 h 6. N	1. 12 NF; 12 TAU; 12 NC 2. 48.25 ± 14.44 vs 54.33 ± 12.67 vs 43.50 ± 13.80 3. Y	HAM-D; BDI-II; CGIS; EQ-5D-5L; SDS; BDNF.	Significant decrease in HAM-D and CGI-S scores vs. TAU (treatment as usual). 58.3% response rate and 50.0% remission rate in the active group. No significant differences in baseline serum BDNF levels among groups.
Wang et al. (2019) [41]	1. ALAY vs high beta down 2. N 3. N 4. 10 5. ? 6. N	1. 24 vs 23 vs 23 TAU 2. 40.330 ± 14.714 vs 42.830 ± 15.816 vs 42.610 ± 13.937 3. Y	BDI-II; BAI.	Significant decrease in BDI-II and BAI scores for both ALAY and beta groups. Both active groups showed similar overall symptom improvement, though the beta group achieved significantly lower BDI-II scores. Decreased parietal high-beta power in the beta group (specifically among patients with comorbid depression and anxiety).
Wu et al. (2024) [46]	1. swLZNFB 2. N 3. N 4. 10 5. 30 minutes 6. N	1. 25 NF vs 23 TAU 2. 37.36 ± 14.08 vs 42.00 ± 14.32 3. Y	EEG; BDI-II; BAI.	Lower scores on psychometric scales in the swLZNFB group versus the control group. Decreased overall EEG abnormalities from pre- to post-test. Decreased current source density of beta1 and beta3 in the prefrontal cortex, anterior and posterior cingulate cortices, and amygdala.
Case series studies				
Peeters et al. (2014) [39]	1. ALAY 2. N 3. N 4. 15–30 5. 34 min 6. N	1. 9 (5 M; 4 F) 2. 46.6 ± 11.7 3. Y	QIDS-SR16; EEG.	Decreased resting-state alpha-asymmetry concurrent with reduced depressive symptomatology. Clinical outcome: 1 responder and 4 remitters out of 9 participants.
Cheon et al. (2016) [43]	1. Left hemisphere beta at F3 with A/T training on Pz 2. N 3. N 4. 16–24 5. 1 h 6. N	1. 20 (4 M; 16 F) 2. 43.00 ± 14.29 3. Y	HAM-D; HAM-A; BDI-II; BAI; CGI-S; EEG.	Significant improvement in clinical symptoms (HAM-D, HAM-A, BDI-II) without significant changes in pre- and post-treatment EEG asymmetry scores.
Case studies				
Kumano et al. (1996) [48]	1. Alpha amplification from O2 2. N 3. N 4. 16+18 5. 24 6. N	1. 1 (M) 2. 37 3. Y	MPI; BDI.	Improved general mood, accompanied by increased alpha activity and decreased parasympathetic activity.
Dias & van Deusen (2011) [45]	1. ALAY and increased Beta/Theta in the left prefrontal cortex 2. N 3. N 4. 10 5. 1 h 6. NO	1. 1 (F) 2. 42 3. N	HAM-D; EEG.	Improvements in emotional profile and self-esteem, though rapid gains during initial sessions suggest a potential placebo effect.
Kaur et al. (2019) [47]	1. 6 z-score training followed by 10 sLORETA NF on anterior cingulate and insula 2. N 3. N 4. 16 5. ? 6. N	1. 1 (M) 2. ? 3. Y	EEG.	Enhanced relaxation accompanied by increased alpha and decreased beta activity, with changes showing a clear linear progression across sessions rather than within individual sessions.

ATQ-P, Automatic Thought Questionnaire-Positive; BAI, Beck Anxiety Inventory; BDI-II, Beck Depression Inventory-II; BDNF, brain-derived neurotrophic factor; CGI-S, Clinical Global Impression-Severity; EEG, electroencephalography; EOG, electrooculogram; EQ-5D-5L, 5-level version of European Quality of Life Questionnaire 5-Dimensional Classification; F, Female; FAS, Fluency Verbal Test; HAM-A, Hamilton Anxiety Rating Scale; HAM-D, Hamilton Depression Rating Scale; M, male; MPI, Maudsley Personality Inventory; N, No; NC, normal control; NFB, neurofeedback; PASAT, Paced Auditory Serial Addition Task; PFT, Phonological Fluency Test; PHQ-9, Patient Health Questionnaire; QIDS-SR16, 16-item Quick Inventory of Depressive Symptoms self-report version; RAVLT, Rey Auditory Verbal Learning Test; SDS, Sheehan Disability Scale; SFT, Semantic Fluency Test; sLORETA, standardized low-resolution brain electromagnetic tomography; SMR, sensorimotor rhythm; ST, Stroop Test; STROOP, Stroop Color-Word Test; swLZNFB, low-resolution electromagnetic tomography Z-score neurofeedback; TAU, treatment as usual; TMT, Trail Making Test; Y, Yes.

## 3. Results

### 3.1 Studies

Of the 11 studies included in this scoping review, all investigated EEG-based neurofeedback for patients with MDD. Six of the studies included control groups: one compared against a psychotherapy placebo, 3 used treatment as usual group, one used a normal control group and one used a normal control and treatment-as-usual group. Two studies were case series and 3 were case studies. Only 2 studies met the criteria for randomized controlled trials. The total number of patients who received active NF was 175. The neurofeedback training time across studies varied widely, ranging from 2.67 hours to 24 hours of total training time.

### 3.2 Neurofeedback Protocols and Training Parameters

The included studies used a variety of EEG-based neurofeedback protocols. Overall, these protocols can be grouped into several main categories: frontal alpha asymmetry training, upper alpha power up-regulation, high-beta down-training, mixed protocols, and Z-score/swLORETA-based approaches, with one study using EEG-driven photic stimulation. Five studies targeted frontal alpha wave, 3 aiming to increase left and/or reduce right frontal alpha activity [38,39,40], one compared it to beta protocol [41] and one targeted upper alpha power over parieto-occipital areas [42]. Two studies employed high-beta down-training [41,43], and three studies used combined protocols, such as sensorimotor rhythm (SMR) or beta training paired with alpha/theta training [43,44,45]. Two studies applied Z-score or swLORETA neurofeedback to target deep brain regions like the anterior cingulate cortex and insula [46,47]. One study used EEG-driven photic stimulation [48]. Session frequency typically ranged from two to three times per week, with 6 to 30 total sessions lasting 20–60 minutes each, using visual or auditory feedback based on real-time EEG signals [38,39,40,41,42,43,44,45,46,47,48].

### 3.2.1 Alpha Protocols

This was the most frequently used protocol, applied in 4 out of 11 studies detailed below:

Choi et al. (2011) [38] conducted a randomized controlled pilot study to assess the efficacy of alpha-wave neurofeedback training in patients with MDD. The study included 24 right-handed participants with MDD, randomly assigned to either an NF training group (n = 12) or a placebo psychotherapy group (n = 11). The NF protocol aimed to enhance right frontal alpha power while reducing left frontal alpha activity, using electrode placements at F3 and F4 (referenced to Cz). Participants underwent 10 NF sessions over five weeks, with two sessions per week [38].

Wang et al. (2016) [40] investigated whether alpha asymmetry neurofeedback (ALAY) could effectively modulate frontal EEG activity and reduce depressive symptoms in patients with MDD. Fourteen patients were randomly assigned to either an NF group (n = 7) or a control group (n = 7). The NF group received six weekly sessions, each lasting one hour, aiming to increase left frontal alpha power (F3) and decrease right frontal alpha power (F4) [40].

Escolano et al. (2014) [42] conducted a controlled study evaluating the cognitive effects of upper alpha neurofeedback training in patients with MDD. The study included 60 participants, comparing an NF group (n = 40) to a non-interventional control group (n = 20). The NF protocol focused on increasing the individual upper alpha power averaged over parieto-occipital locations using electrode placements at P3, Pz, P4, O1, and O2. The NF group underwent a total of eight training sessions over four weeks, with each session comprising five 4-minute trials for a total of 20 minutes of active training per session [42].

Peeters et al. (2014) [39] conducted a case series study to evaluate the feasibility and effectiveness of alpha asymmetry neurofeedback in MDD. Nine participants received up to 30 NF sessions over a 10-week period. The electrode placement was at F3 and F4. Participants were required to maintain stable antidepressant use for at least six weeks prior to and throughout the study to control for medication effects [39].

### 3.2.2 Alpha vs. Beta Protocols

The study conducted by Wang et al. (2019) [41] compared the effects of two neurofeedback protocols alpha asymmetry training and high-beta down-training with treatment as usual (TAU) on depression and anxiety symptoms in 47 patients with MDD and comorbid anxiety. Participants were divided into a TAU (n = 23) group and two NF groups: the ALAY group (n = 24) (F3 and F4) and the Beta group (n = 23) (P3 and P4), which targeted high-beta reduction. Both NF groups received 10 sessions, conducted twice weekly over five weeks [41].

### 3.2.3 EEG-Driven Photic Stimulation

Kumano et al. (1996) [48] presented a case study exploring the potential of EEG-driven photic stimulation. The intervention targeted a 37-year-old male patient and involved photic stimulation synchronized with his alpha rhythm at O2 (referenced to A1), aiming to enhance alpha wave activity and stabilize mood. Treatment was delivered in two cycles of 16 and 18 sessions during an inpatient program [48].

### 3.2.4 Mixed Protocols

Lee et al. (2019) [44] conducted an open-label pilot study to explore the benefits of neurofeedback as an augmentation therapy in treatment-resistant depression (TRD). The study included 12 TRD patients in the NF group, 12 TAU patients and 12 normal controls. The NF group received 12 to 14 sessions over 12 weeks, with each session consisting of 30 minutes of sensorimotor rhythm (SMR) (T4) or beta training (F3 or T3) followed by 30 minutes of alpha/theta training at Pz [44].

Cheon et al. (2016) [43] used a dual-protocol approach targeting frontal beta activity and alpha/theta training. The study included 20 patients with MDD who underwent 16 to 24 sessions over eight weeks, attending two to three sessions per week. Each session included 30 minutes of beta training at F3 and 30 minutes of alpha/theta training at Pz [43].

Dias & van Deusen (2011) [45] introduced a unique neurofeedback protocol designed to reduce depressive symptoms through a multi-target approach. The protocol aimed to increase alpha asymmetry to the right, increase the beta/theta ratio in the left prefrontal cortex, and reduce beta-3 activity across the prefrontal cortex at the same time. Electrodes were placed at F3 and F4. The study involved a single patient who received 10 one-hour NF sessions [45].

### 3.2.5 Z-Score and swLORETA Neurofeedback

Wu et al. (2024) [46] examined the effects of Z-score–based swLORETA neurofeedback in 25 MDD patients compared to 23 patients receiving treatment as usual. Participants received 10 NF sessions over five weeks, with two sessions per week. Training targeted deep brain regions involved in emotional regulation, including the prefrontal cortex (PFC), anterior cingulate cortex (ACC), posterior cingulate cortex (PCC), and amygdala. The protocol used a computer-driven selection of the 1580 most deviant EEG parameters, aiming to normalize them to a Z-score of 0 [46].

Kaur et al. (2019) [47] described a case study investigating the impact of standardized low-resolution brain electromagnetic tomography (sLORETA) neurofeedback on depressive symptoms and EEG biomarkers. One participant with clinically significant depressive symptoms underwent 16 NF sessions: six sessions of surface Z-score training followed by 10 sessions of sLORETA-based training targeting the insula and ACC. Each session lasted 20 minutes, administered three times per week [47].

### 3.3 Effects on MDD

Treatment outcomes were reported for 175 patients across 11 studies, all of which noted preliminary reductions in depressive symptoms following EEG-based neurofeedback (NF). Across studies, some changes were observed regardless of protocol type, although some differences in symptom domains were noted (synthetized in Table 1). Frontal alpha asymmetry protocols used by Choi et al. (2011) [38], Wang et al. (2016) [40], Peeters et al. (2014) [39], and the ALAY group in Wang et al. (2019) [41] were frequently associated with short-term trends toward improvements in depression scores. For example, Choi et al. [38] observed a significant decrease in HAM-D scores (11.33 ± 7.52 to 4.08 ± 4.14; F(1, 20) = 5.96, *p* < 0.05) and BDI-II scores (22.75 ± 12.35 to 9.08 ± 6.92; F(1, 20) = 6.87, *p* < 0.05) from baseline. In contrast, Wang et al. (2016) [40] reported a reduction in BDI-II scores from 35.75 to 29.25 (t = 1.32, *p* > 0.05) after six sessions in the responder group. Peeters et al. (2014) [39] found that 4 of 9 patients achieved remission, supported by concurrent EEG asymmetry normalization.

High-beta down-training, applied in Wang et al. (2019) [41] and Cheon et al. (2016) [43], showed preliminary effects, especially in anxious subgroups. In Wang et al. [41], the Beta group had significantly greater anxiety reduction than the ALAY or TAU groups. Cheon et al. [43] reported that more than half of participants reached remission, with marked improvement in depressive symptoms over 20 sessions [41,43].

Cognitive changes were reported by Choi et al. [38], Wang et al. [41], Escolano et al. [42] and Lee et al. [44]. However, only Choi et al. [38] and Escolano et al. [42] employed dedicated cognitive assessments. Choi et al. [38] observed initial improvements on the Semantic Fluency Test (SFT) (39 ± 5 to 45 ± 2; F(1, 21) = 9.45, *p* < 0.01), the Phonological Fluency Test (PFT) (45 ± 3 to 55 ± 2; F(1, 21) = 4.02, *p* < 0.1), and the Stroop Test (ST) (congruent: 700 ± 25 to 630 ± 31; incongruent: 770 ± 30 to 685 ± 50; F(1, 21) = 4.98; *p* < 0.05). Escolano et al. [42] measured cognitive performance before and after the intervention, reporting a statistical significant cognitive transformation in working memory and processing speed on the PASAT test for the NF group (errors decreased from a mean of 13.45 ± 1.09 to 10.13 ± 1.08, *p* < 0.001; time decreased from 253.23s ± 13.25 to 213.68s ± 12.13, *p* < 0.001), while the control group showed no significant change. The NF group also exhibited statistically significant within-group improvements in episodic memory RAVLT recognized words (12.25 ± 0.43 to 13.15 ± 0.30, *p *= 0.002), executive functions TMT part B (101.50 ± 14.57 to 84.87 ± 11.91, *p* = 0.007), and verbal fluency FAS evoked words (43.74 ± 1.87 to 46.85 ± 1.90, *p* = 0.013). Furthermore, a positive correlation was found between this improvement in processing speed and an increase in beta power [42]. In contrast, Lee et al. [44] and Wang et al. [41] did not use specific cognitive measures, instead relying on HAM-D and BDI-II total scores, with cognitive aspects described only narratively. Effect sizes were not explicitly reported by the authors of the included studies. Given the scoping nature of this review and the small sample sizes across studies, post hoc calculations of effect sizes could be statistically unreliable and might overstate clinical efficacy.

Advanced swLORETA and Z-score NF, featured in Wu et al. (2024) [46] and Kaur et al. (2019) [47], targeted deep brain regions such as the anterior cingulate cortex and insula. Wu et al. [46] observed significant symptom reduction and enhanced network connectivity post-training. Kaur et al. [47] reported improved depressive symptoms alongside normalization of EEG waves in a treatment-resistant patient.

Kumano et al. (1996) [48] offered a unique EEG-driven photic stimulation protocol, leading to improved mood stability and autonomic regulation in a single inpatient case.

### 3.4 Long-Term Efficacy and Follow-Up Assessments

Long-term efficacy and follow-up assessments were limited in the included studies. Only Choi et al. (2011) [38] reported follow-up data, with a one-month follow-up period during which participants maintained symptom reductions, suggesting potential lasting effects of neurofeedback on mood regulation. The other studies either lacked follow-up or assessed outcomes solely immediately post-treatment, preventing conclusions about the durability of neurofeedback’s effects.

Overall, the absence of follow-up assessments in the vast majority of studies represents a key gap in the current literature. Given its importance for evaluating sustained clinical benefits, this limitation may be more appropriately emphasized in the Discussion section.

## 4. Discussion

This scoping review maps the available literature on electroencephalography-neurofeedback interventions in MDD, with attention to reported protocol characteristics, neurophysiological targets, and outcomes. By limiting inclusion to EEG-based neurofeedback, the paper seeks to clarify methodological features that may be obscured in broader syntheses combining heterogeneous biofeedback and neurofeedback approaches [30,49]. Rather than evaluating effectiveness, this scoping review describes how EEG-NF has been applied across studies and highlights areas where evidence remains limited or inconsistent, which may constrain interpretation, replication, and future clinical application. The key findings that emerged are: there is substantial heterogeneity in EEG-NF protocols, most studies report short-term symptom improvement across diverse approaches without a clearly superior protocol, and follow-up and controlled designs are consistently lacking, limiting conclusions about the durability and specificity of effects.

As this study was conducted as a scoping review, no formal risk of bias assessment was performed. This limits the ability to evaluate the internal validity of included studies and should be considered when interpreting the findings. Furthermore, the inclusion of lower-level evidence, such as case reports and case series, while appropriate for capturing the pulse of emerging research in a scoping review, may increase the bias and limit the generalizability of findings.

A central finding of this scoping review is the substantial heterogeneity of EEG-NF protocols employed in MDD research. Beyond simply cataloging these approaches, it is important to consider their hypothesized mechanisms and clinical applicability. The most frequently utilized approach, frontal alpha asymmetry training, is largely based on the approach-related affect and emotion regulation [50,51]. Grounded in the premise that alpha power is inversely correlated with cortical activation, these protocols theoretically aim to decrease left frontal alpha power (at F3) to promote approach-related positive affect, while increasing right frontal alpha power (at F4) to mitigate negative, withdrawal-related emotions. While a notable strength of this protocol is its established theoretical framework in emotion regulation, its clinical translation appears inconsistent. Although several studies report concurrent reductions in depressive symptoms, the actual degree of EEG modulation and its direct correlation to clinical improvement vary considerably [38,39,40,41]. These observations may indicate that frontal alpha asymmetry is not clearly associated with depressive individuals and clinical improvement, suggesting that alpha asymmetry might act more as a state-dependent marker rather than a universally viable therapeutic target [50,52], underscoring the need for careful interpretation and patient stratification in future MDD interventions, uncertain, highlighting the need for more standardized approaches and mechanistically informed protocol selection.

Beyond frontal alpha asymmetry, several studies explored alternative EEG-NF approaches targeting distinct symptom clusters in MDD, revealing a divergence between cognitive and emotional therapeutic goals [53]. For instance, Escolano et al. [42] utilized upper alpha up-regulation over parieto-occipital areas specifically to target cognitive impairments, demonstrating a improving working memory and processing speed compared to a non-interventional control group.

Beta protocols, such as beta up-training in left dorsolateral prefrontal regions, were primarily grounded in models of executive dysfunction, aiming to improve impaired cognitive control and attention [43]. In contrast, theta modulation and alpha/theta training, often conducted at parietal or midline sites, focused heavily on emotional outcomes. These protocols sought to reduce negative emotional processing, stabilize mood, and promote relaxation by shifting arousal states [44,54,55,56]. Some interventions bridged these domains through multi-component protocols combining theta/beta modulation with alpha asymmetry or beta down-training [45], or by utilizing connectivity-based swLORETA neurofeedback and individual alpha-synchronized photic stimulation to target deep cortical and limbic structures implicated in both cognitive control and emotion regulation [47,48]. Collectively, this dual focus on cognitive versus emotional outcomes underscores the absence of a unifying theoretical framework in EEG-NF design, highlighting how different frequency targets are leveraged to address the multifaceted, heterogeneous nature of depression.

More recent investigations expanded beyond surface EEG features [57,58] to connectivity-based and source-localized approaches using low-resolution electromagnetic tomography and Z-score neurofeedback [46,47]. These methods targeted deeper cortical and subcortical regions implicated in emotion regulation and cognitive control, including the anterior cingulate cortex, insula, and prefrontal networks. Interestingly, source-localization analysis by Escolano et al. [42] demonstrated that even surface-level upper alpha training could induce current density increases in deep subcortical regions, specifically the subgenual anterior cingulate cortex (sgACC) in the alpha band, highlighting an anatomical overlap in cognitive and affective processing substrates.

While these studies represent an important methodological evolution in the field, their limited number and reliance on specialized equipment constrain broader generalizability [59]. From a scoping perspective, these findings illustrate an emerging trend toward network-level neuromodulation rather than isolated frequency-band training.

Training frequency and duration varied substantially across studies, typically ranging from 10 to 30 sessions of 30–60 minutes, with longer interventions showing higher remission rates in some reports [39]. However, the absence of consistent long-term follow-up data limits conclusions regarding the durability of effects. Overall, no single protocol emerged as superior, highlighting the need for personalized, biomarker-informed NF approaches and for larger, controlled comparative studies to support protocol standardization in clinical practice [60].

Across the mapped literature, study designs were predominantly exploratory, with small sample sizes and a mix of randomized, non-randomized, and case-based methodologies. Outcome measures varied widely, encompassing clinician-rated depression scales, self-report questionnaires, cognitive performance metrics, and EEG-derived neurophysiological indices. This variability limits direct comparison across studies and complicates efforts to synthesize outcomes quantitatively.

Notably, long-term follow-up assessments were rarely included. Only a small number of studies evaluated whether symptom reductions or EEG changes persisted beyond the immediate post-intervention period, leaving the durability of EEG-NF effects largely unexplored. This represents a significant gap in the evidence base, particularly given that sustained symptom remission is a central goal of depression treatment. Importantly, the near-complete absence of longitudinal follow-up data limits understanding of whether observed clinical and neurophysiological improvements translate into sustained therapeutic benefits over time. As such, the durability of effects remains one of the most under-investigated aspects of EEG-NF in MDD.

A prominent methodological issue identified in this scoping review is the limited use of sham or active control conditions. Given the interactive and engagement-driven nature of neurofeedback, expectancy effects, motivation [61,62], and non-specific therapeutic factors may contribute to observed improvements. While this scoping review does not aim to evaluate efficacy or placebo effects, the scarcity of controlled designs highlights an important limitation in the current research landscape [26,61,63].

Blinding procedures were inconsistently reported, and EEG acquisition, referencing, and preprocessing methods varied substantially across studies. These inconsistencies further limit reproducibility and underscore the need for standardized reporting guidelines in EEG-NF research.

By mapping the existing literature, this scoping review identifies several priorities for future investigation. First, greater standardization of EEG-NF protocols, including electrode placement, frequency targets, session number, and training thresholds, would enhance comparability across studies and support cumulative knowledge building. Second, larger, adequately powered studies incorporating sham-controlled or comparator conditions are needed to better characterize the specific contribution of EEG-NF relative to non-specific effects [26,61], Third, longitudinal designs with extended follow-up periods are essential to determine the persistence of both clinical and neurophysiological changes.

Finally, emerging evidence suggests that individual baseline EEG characteristics may influence responsiveness to specific neurofeedback protocols. Future research may benefit from stratifying participants based on pre-training biomarkers, thereby advancing personalized EEG-NF approaches rather than one-size-fits-all interventions [64].

## 5. Conclusions

This body of literature indicates that neurofeedback has been explored as a potential intervention for individuals with Major Depressive Disorder, with studies reporting modulation of EEG activity alongside improvements in depressive symptoms, emotional regulation, and aspects of cognitive functioning. However, the evidence base is characterized by substantial heterogeneity in study design, neurofeedback protocols, outcome measures, and patient populations, as well as limited use of sham-controlled conditions. As a result, definitive conclusions regarding clinical efficacy cannot yet be drawn.

Future research should prioritize the standardization and transparent reporting of NF protocols, the identification of neurophysiological and clinical predictors of treatment response, and the systematic evaluation of NF as an adjunct to pharmacological and psychotherapeutic interventions. Furthermore, the scarcity of long-term follow-up assessments highlights an important gap in the literature, underscoring the need for longitudinal studies to clarify the durability and clinical relevance of NF-related changes in MDD.
